# Introduction: Spatial Perspectives and Medical Humanities

**DOI:** 10.1007/s10912-014-9319-z

**Published:** 2014-12-09

**Authors:** Sarah Atkinson, Ronan Foley, Hester Parr

**Affiliations:** 1 Department of Geography and the Centre for Medical Humanities, Durham University, Lower Mountjoy, Co. Durham, DH1 3LE UK; 2 Department of Geography, National University of Ireland, Maynooth, Co. Kildare Ireland; 3 Geographical and Earth Sciences, University of Glasgow, East Quadrangle, Glasgow, G12 8QQ UK

The scope and purpose of the medical humanities have been taking exciting new directions in recent years. The pedagogical roots of the field positioned medical humanities as supplementary to the scientific underpinnings of medicine in bringing humane judgement, insight and resource to the medical practitioner (see Brody 2009; McManus 1995). But new engagements with a range of critical, cultural and social theories have brought with them new encounters by scholars in the medical humanities with what may properly constitute the objects and categories of their enquiries.

One direction taken by such new engagements concerns the understandings of what it means to be human, understandings which for the practice of medicine ‘require an atomistic view of how human beings work as physically embodied individuals and as persons within their social context’ (Macnaughton 2011, 927). As the medical humanities open to new conceptualisations of being human, the nature of embodiment and of social context highlighted by Macnaughton demand greater interrogation. A second direction of new engagements concerns the understandings of what constitutes health, ill-health, disease and demands attention to the policies, sites and cultural processes through which health and ill-health are both produced and defined. This move includes recognition of non-medical, non-clinical sites of health and illness (see Colls and Evans 2008), the historical trajectories that may inform and challenge contemporary assumptions of various medical categories (see McKinstry 2012) and the different intersecting ‘scales’ of health and illness (see McKenna 2012). And underlying much of these new directions is a more acute and overt critical politics of health and illness emerging through the medical humanities (see Gilman 2011).

Given these various new directions, it is perhaps surprising that the field of social and cultural geographies has garnered limited attention from the medical humanities. Much of the trans-disciplinary material identified as medical humanities contains within it a strong sense of the spatial. Work drawn upon from history, art, literature, even music may have identifiable locations, whether as the subjects or objects of research and research with specific clinical and applied expression is usually set in real places: hospitals, clinics or homes. However, there is a rich and growing body of research across social, cultural and health geographies that makes space for and foregrounds place in much more explicit ways and the situated nature of being and becoming urgently require the theoretical insights of those who specifically focus on the nature of space and place.

This special issue of six papers has emerged from one endeavour both to redress the neglect of Human Geography within medical humanities and to draw geographers into dialogue with the medical humanities. Geography is one of the foundational departments to the current Centre for Medical Humanities at Durham University in the United Kingdom. As such, the Centre has had involvement in a number of conferences, workshops and seminars together with geographers and has hosted visiting scholars from the geography discipline. In 2011, at the 14th International Medical Geography Symposium held at Durham University, geographers and medical humanities scholars came together in two sessions dedicated explicitly towards building this conversation. The broad thematic intent was to look more closely at and uncover ongoing work on the potential role for Geography within the wider Medical Humanities and in particular to ‘emplace’ the subject more fully.

The six papers offer pieces for reflection from both geographers and those self-identifying as medical humanities scholars. The pieces pick up the themes of those new directions in medical humanities indicated above in the attention variously given to embodiment, non-clinical spaces and activities for health and well-being and the broader considerations of scale and politics. These general trends in medical humanities are cross-cut with contributions to theoretical debates on energy, space, time, movement and affect.

The opening paper by Ronan Foley explores the nature of informal or folk medicine at a time in the mid-19th century when formal medicine was gaining ascendancy. Foley draws on an historical methodology familiar to the medical humanities, mining archives for traveller accounts and surveys of folklore and healing practices. Through these, Foley can interrogate the connections between the two forms of medical practice unusually from the local perspectives of those directly involved in informal folk medicine. He indicates a far more subtle set of hybrid and fluid relations between informal and formal medicine situated into the cultural setting of beliefs and practices around the body. Analysis of these narratives highlight the ways in which folk cures were often specific to either places, seasons and/or objects which thus constitute sites, in time and space, from which energies could be drawn for the ailing body as a body specifically situated in place. Energies, flows of energy and oscillations in energy emerged as central to much of the understanding of cure as a process of reinvigoration.

Geraldine Perriam’s paper continues the exploration of specific sites of healing as embedded within the culture and beliefs of particular times and places. Geographer Wil Gesler introduced the notion of therapeutic landscapes into the lexicon of scholarship in the late 1990s (see Gesler 1992). The concept has proved itself useful in examining the multi-layered relationalities that structure senses of healing, places and the experiences of those inhabiting these. Perriam draws on this body of work with particular attention to the category of the spiritual through two examples of healing sites, a centuries-old shrine and a more recent retreat. The narrative histories of the sites, old and new, are similar in identifying a productive performative relationship with health whilst also tapping into older ascribed energies of holy places, suggesting how place, faith and informal or complementary medicine is co-produced in place. Whilst both of Perriam’s sites have Christian roots, her elaboration of spirituality is not limited to a narrow, faith-based definition but allows a broader comprehension of moral orientation and sense of purpose. Healing is then conceived as requiring a (re-) integration of that which has become fragmented, a return to wholeness and unity of body, mind and spirit. Perriam indicates how neglect of the ‘spiritual’, as broadly conceived, neglects an integral part of both the historical and contemporary encounters with healing space and their flows of energy.

The importance of the spiritual in contemporary health practices is echoed in the paper by Chris Philo and colleagues which draws on a case study of ‘new spiritual practices’ of the self (citing Heelas 2008) and, in particular, of yoga and meditation. As in Foley’s historical narratives of healing, the contemporary first person accounts in this paper feature energies and energy flows. The study positions these new spiritual practices as an embedded part of busy modern lives and not, as has been implied, a practice of separation or retreat from those lives. Philo et al., offer a vision of ‘energy geographies’ that animates lifeworlds, energy flows and their affectivities in the contemporary pursuit of well-being. Whilst positioning the study as geographies of energy/energy geographies, the paper also offers a stimulating research agenda for the medical humanities.

Martyn Evans engages the notion of human energy rather differently, through a non-geographical lens as a philosopher. He argues for an imaginative energy questing for a sense of wonder that has the capacity to transfigure our comprehension of the world around us. Evans’ argument builds on Arthur Frank’s writing on wonder in which the implicit value of place within that discussion slowly, even reluctantly, emerges. The role of wonder is exemplified by Evans in considering the role of the patient in a set of encounters in medical space in which embodiment, emotion and presence all play a constant cognitive, if often unexpressed, form. Evans is also usefully resistant to the potential value of place in ways that are of critical value. And Evans’ core philosophical concern with the mental elusiveness of wonder and the workings of the human mind suggest a dualism in which the mind’s ability to wonder causes it to wander, away from space and the territoriality of the body.

The idea of transfiguration has resonance with the fifth paper in this special issue. Mike White and Mary Robson offer a reflection on how community-based arts and health projects may be beneficial to those involved through two case studies of lantern parades. These parades are annual events, a defined and limited moment in time and space that is both grounded in a locality and mobile in passing through that space. The preparatory work in making the lanterns for the parades bring together different constituencies in the local area, constituencies that may have had little previous contact, may be suspicious or even overtly hostile to one another. The parade itself traverses the neighbourhood engaging participants and spectators alike into what, through annual repetition, becomes a local ritual. White and Robson describe their involvement in the two case studies over many years and reflect on how lantern parades may contribute to well-being via social capital, the gift, mutual exchange and ritual. Participants talk of belonging, of safety, of inclusion and perhaps most importantly, of seeing differently in a transfigured vision and imaginary of both place and people. And central to these potential gains is the energy of light.

The final paper engages the dialogue between medical humanities and geography in a more explicit manner. Sarah Atkinson and colleagues working across both geography and medical humanities in the Durham Centre engage a debate within the field of medical humanities around the terminology of medicine and health. Atkinson et al. examine the unrolling of a similar debate that has already occurred within the social sciences through the specific example of the geographies of health. The addition to the field of medical geographies of a politically framed cultural field of geographies of health was associated with the development of distinct and divergent forms of intellectual endeavour. This divergence left important lacunae in research topics and underpinning assumptions at the interface of those areas seen as ‘medical’ and those captured under ‘health’. Critical and cultural geographers of ‘health’ are now revisiting and re-valuing the categories of the ‘medical’ and of ‘health’ to redress this false divergence. The special issue thus concludes with an explicit offering from the experiences of different pathways within the geographies of medicine and health to the future directions in the field of medical humanities.

The collection offers a number of reflections relevant to the field of medical humanities. Central to the empirical papers is the concept of energy, human energies, flows of energies, affective energies and perhaps spiritual energies. These papers offer explorations of health, healing or transfiguring wonder that are situated in non-clinical settings and make explicit the connections of space, place and time. A range of methodological approaches are evident, using historical archives, participant observation, interviews, diaries and existing literature. Perhaps most importantly, human geography is envisioned through this work as a bridging discipline across physical, social, cultural and medical concerns, and working with hybrid concepts and dialogues. We hope this special issue will be the first of many future conversations between the medical humanities and human geography in exploring the ‘worlding’ of health, healing and well-being.
